# A 1980–2018 global fire danger re-analysis dataset for the Canadian Fire Weather Indices

**DOI:** 10.1038/sdata.2019.32

**Published:** 2019-02-26

**Authors:** Claudia Vitolo, Francesca Di Giuseppe, Blazej Krzeminski, Jesus San-Miguel-Ayanz

**Affiliations:** 1 European Centre for Medium-range Weather Forecasts, Reading, UK; 2 European Commission, Joint Research Centre, Ispra, Italy

**Keywords:** Natural hazards, Environmental chemistry

## Abstract

This data descriptor documents a dataset containing over 38 years of global reanalysis of wildfire danger. It consists of seven fields to assess fuel moisture as well as fire behavior. The methodology employed to generate these data is based on the Canadian Forest Fire Weather Danger Rating and utilizes weather forcing from ERA-Interim, a global reanalysis dataset produced by the European Centre for Medium-range Weather Forecasts. Global fire danger reanalysis data are used to quantify the climatological expectation of fire danger at a certain time of the year and for any location on the globe. It can be regarded as a complementary product to the fire danger forecasts issued daily by the Global Wildfire Information System (GWIS) under the umbrella of the European Copernicus program.

## Background & Summary

Forest fires are extremely costly hazards in terms of lives, natural resources and infrastructures. In the last decades, the low predictability of ignition due to anthropogenic factors have increasingly put a strain on forestry agencies^1^ as well as on local economies. According to NOAA’s National Center for Environmental Information, the costs from the 2017 wildfire season in western USA were $18 billion, triple the previous U.S. annual wildfire cost record^2^. Wildfires are also recognised as one of the most significant environmental threats in Europe, where most of the burnt areas are concentrated in the Mediterranean region^3^.

The scientific community is actively studying (through *in situ* observation and remote sensing) fire behavior to better identify the atmospheric predictors that determine a sustained fire activity once an ignition has occurred. These predictors are then used to assess current danger condition and to forecast its evolution in the future. It is intuitive that low humidity combined to high temperature tends to dry dead and live fuels which ignite easier than moist fuel. In addition, high wind variability favors the spread over larger areas, compared to cases in which wind blows mainly in one dominant direction. Most of these mechanisms are in-built in the Canadian Fire Weather Index (FWI) system^4,5^ which is one of the most widely used model to estimate fire danger worldwide^6,7^. The FWI operates by predicting the responses of fuel moisture to atmospheric forcings at different soil depths and by combining these to derive fire behavior indices in terms of ease of spread and intensity. The FWI danger rating system was designed to exploit the information provided by in situ observations and it only depends on weather variables. Therefore, it only provides a qualitative overview of where conditions could lead to uncontrollable fires. However, by lacking information on the available fuel load, the vegetation stage and the topography (e.g. terrain slope) FWI is often used in combination to local information to gain a quantitative assessment of fire activity once an ignition has taken place. For its simplicity of calculation the FWI danger rating system is adopted by many fire agencies around the world and operationally used to issue fire alerts and allocate resources on the ground. The FWI system is also an important building block of the Global Wildfire Information System (GWIS, http://gwis.jrc.ec.europa.eu/), a joint initiative of the GEO (http://www.earthobservations.org) and the Copernicus (http://copernicus.eu/main/emergency-management) Work Programs, which provides access to worldwide information on wildfires. Information about the current situations can be obtained from the GWIS’ web-based viewer which is designed for efficient browsing of fire danger forecasts, as well as information on ongoing active fires, assessment of burnt areas and fire emissions. The daily forecasts of FWI model outputs are provided by the European Centre for Medium-range Weather Forecasts (ECMWF) using the atmospheric forcings calculated from its ensemble prediction system (with a 10-day time horizon).

Although real-time fire danger forecasts are fundamentally important for first responders, it is also important to inform users of the value ranges considered dangerous for a given area. Indeed, the FWI was designed as a regional danger rating system and a meaningful interpretation of the FWI values can only come from the comparison with the range of possible historical values in that area. This historical information is very valuable and it is encapsulated in the reanalysis records presented here^8,9^. A reanalysis dataset is generated by combining a numerical weather prediction model and quality-controlled observations for past conditions in a statistically optimal way by means of an assimilation scheme^10^. As the final output is mostly controlled by observations, to a large extent it can be considered as a good proxy for observed meteorological conditions. When compared to point-wise observations it also has the benefit to provide spatially consistent fields, also in regions with poor observational coverage. The consistency in time and space of a reanalysis dataset makes it the ideal choice to investigate temporal trends and changes in the spatial variability of fire behavior. It is also a valuable resource to investigate the relation between forest fires and climate variability over large areas. These applications are deemed particularly relevant as the occurrence, length and frequency patterns of fire events are likely to change in a changing climate^11^ and favorable fire conditions are going to be exacerbated by raising temperature^12,13^. To ease the exploitation of this dataset ECMWF has also developed an open-source R package, called ‘caliver’^9^. This package has built-in functions to manipulate fire danger reanalysis data, for example by spatial area cropping and by merging data slices to build timeseries. In the ‘Usage Notes’ section we provide examples of the sort of manipulations that can be performed using the caliver package.

Over time, users’ requests for reanalysis data has increased and, in order to efficiently respond to users’ needs, ECMWF has started to archive reanalysis of the FWI system on Zenodo, a research data repository which assigns all publicly available uploads a Digital Object Identifier (DOI) to make the upload uniquely citeable. This repository is updated regularly and versioned according to the stable releases of the ECMWF fire danger model. This paper describes the FWI reanalysis dataset, an extremely valuable resource for forestry agencies and scientists in the field of wildfire danger modeling. We hope that this work will boost the data uptake and visibility in the wider scientific community and beyond.

## Methods

The fire danger reanalysis dataset is made of seven gridded fields (or indices) calculated from the Canadian Fire Weather Index model using weather forcings from the ECMWF ERA-Interim reanalysis dataset^8,20^. Fire danger indices are routinely computed and archived on an internal ECMWF database in NetCDF format, packaged in daily layers and compressed to facilitate transfer over the network. At the time of writing, data are available from 1st January 1980 to 30th June 2018 with a daily time step at 12 local time. Layers are defined on a regular grid in the World Geodetic System 1984 (also known as WGS 1984, EPSG:4326). Latitudes span the range from −90 to +90 degrees, while longitudes are in the range from 0 to 360 degrees. The spatial resolutions is 0.7 degrees (about 80 Km), thus each file is made of 256 rows, 512 columns and 14061 layers (one layer per time stamp).

In their raw form, the layers are defined in a coordinate system common to other ECMWF products but requires manipulation in order to be combined with other information (e.g. administrative boundaries and satellite imagery). In addition, there is a fair amount of pre-processing needed to make the data in a form that is suitable to analyze historical trends and verify spatial patterns using remotely sensed observations, as described in a previous work^9^. For these reasons, the FWI indices documented here and archived in Zenodo are a slightly modified version of what is currently available on the ECMWF archive. The processing workflow involved: (i) downloading and de-compressing daily raster maps for the above mentioned seven fields, (ii) rotating each raster map so that longitudes are defined in the range [−180, +180] and (iii) stacking the rotated raster maps of each index together in a three-dimensional NetCDF file. Data transfer, or stage (i), would be the most time consuming part of the workflow, especially if done by external users. Stage (ii) ensures consistency with conventions adopted by other sources of relevant information such as: burned areas products from the Global Fire Emission Database (GFED4, http://www.globalfiredata.org) and administrative boundaries provided by the Global Administrative Areas database (GADM, https://gadm.org/). Lastly, stage (iii) generates a data cube of about 7 GB for each fire danger index. This may sound a large dataset, but modern computers and libraries can handle this volume of information very efficiently^9,14^.

### Code availability

In the spirit of reproducibility, the code to extract the data from the ECMWF archive (using internal access credentials), apply the changes described in the methods section and run the validation analysis, is openly available in the GEFF-ERAI repository on GitHub^15^.

## Data Records

The FWI rating system is described in detail by Van Wagner^5^. Below the fire danger indices made openly available by ECMWF are briefly described, including links to the related archives on Zenodo. Due to the large file size of each index (about 7 gigabytes), these are archived separately. The version is updated, at least, once every year. Updates consist of appending data for the previous year to the existing records or a re-analysed dataset if any bug was found and fixed. Every DOI mentioned below represents all versions of each dataset (v2.2 is the most up-to-date at the time of writing), and the link will always resolve to the latest one.

The dataset contains three measures of soil moisture calculated with different codes depending on the fuel consistency:

- The **Fine Fuel Moisture Code** (FFMC, Data Citation 1) is a numeric rating of the moisture content of litter and other cured fine fuels occupying the first fuel bed layers (surface layer, 1-2 cm deep). This code is an indicator of the relative ease of ignition and the flammability of fine fuel. FFMC is characterised by a fast response to weather variations, with a timelag of 2/3’s of a day under standard conditions. The FFMC rating is defined in the range from 2 to 101, as suggested by Van Wagner^5^. The typical FFMC default startup value is, by convention, equal to 85. The reanalysis dataset considered here is obtained as a single model run starting on 1st January 1979. However, the first year is discarded to ignore the effects of the initial model spin-up and no seasonal start-stopping rule is implemented (same applies to the other indices).
- The **Duff Moisture Code** (DMC, Data Citation 2) is a numeric rating of the average moisture content of loosely compacted organic layers of moderate depth (duff layer, 5–10 cm). This code gives an indication of fuel consumption in moderate duff layers and medium-size woody material. DMC is characterised by a medium-term response (about 10–12 days) to weather variations. The rating is defined in the range from zero to infinity, however, the default startup value is conventionally set equal to 6.
- The **Drought Code** (DC, Data Citation 3) is a numeric rating of the average moisture content of deep, compact organic layers (deep duff layer, 1–20 cm). This code is a useful indicator of seasonal drought effects on forest fuels and the amount of smoldering in deep duff layers and large logs. DC has a long-term response (about 50 days) to weather variations. The rating is defined in the range from zero to infinity, with a default startup value equal to 15.

From these weather-driven fuel moisture calculations, the FWI model generates the following fire behavior indices:

- The **Initial Spread Index** (ISI, Data Citation 4) is a numeric rating that measures the rate at which a fire would spread in its early stages shortly after ignition. It combines the effects of wind and the FFMC on rate of spread without the influence of variable quantities of fuel. ISI is defined in the range from zero to infinity.
- The **Build Up Index** (BUI, Data Citation 5) is a numeric rating of the total amount of fuel available for combustion. It is a weighted combination of the DMC and DC and defined in the range from zero to infinity.

Lastly, the model computes the following two indices:

- The **Fire Weather Index** (FWI, Data Citation 6) integrates current ISI and BUI to produce a unitless index of general fire intensity. It is defined in the range from zero to infinity and it is suitable as a general index of fire danger.
- The **Daily Severity Rating** (DSR, Data Citation 7) is a numeric rating of the difficulty of controlling fires. It is a non-linear transformation of the Fire Weather Index, intended to be more directly proportional to the expected effort required for fire suppression. It is also considered suitable as fire weather measure to be averaged over space (i.e. region) and time (i.e. season).

All datasets are calculated using a daily time step by interpolating the atmospheric fields at local noon when fire conditions are considered to be at their worst. If a pixel is covered in snow for more than 20% of its area, the values of ISI, BUI and FWI are reset to zero. This is to acknowledge that in snow areas fire risk should be considered null. However the calculation of the fuel moisture codes (FFMC, DMC, DC) is not suspended. This implies that there is no a priori overwintering applied as the decision on the length, start and stop of the fire season is left to the user.

Each index describes a different aspect of the effect that fuel moisture and wind have on fire ignition probability and its behavior, if started^5^. They can therefore have different fields of applications. However it is worth stressing that these datasets, while being largely employed in fire control and management, do not provide any indication on where a fire is likely to strike. In the calculation there is no information about the likelihood of an ignition which, being either human or natural in origin, remains a stochastic highly unpredictable process.

## Technical Validation

The validation procedure consists of (i) assessing that the implemented algorithm provides numerical values consistent with what given by other FWI available codes (ii) assessing the quality of the dataset as a proxy for observed values by comparing it with the FWI calculated using local data from a set of monitoring stations. The former is carried out by comparing the results of the ECMWF code and the output of the R package *cffdrs*^16^. The latter, instead, is obtained by point-inspecting the FWI data cube stored in Zenodo using the coordinates of a representative set of manned and automated land-based weather stations usually referred to as SYNOP stations.

### Algorithm validation

An R package called *cffdrs*^16^, for the calculation of FWI system indices, is available through the official CRAN repository. We feed both ECMWF model and this package with the same observed input data records and check the consistency of the outputs (see Table 1). The test values are as follows:

- latitude = 45.98 degrees,
- temperature = 17 C,
- relative humidity = 42,
- wind speed = 25 Km/h,
- precipitation = 0 mm

Although the outputs are rather close, they do not match exactly. The reason is that ECMWF model follows the formulation defined in the reference FWI implementation outlined in Van Wagner^17^ without modifications. Wang *et al.*^16^, instead, have modified some of the original equations (i.e. eqs 12 and 15) leading to the calculation of DMC and DC. As a consequence, FWI and DSR also slightly differ.

### Comparison with observed FWI

The last part of the validation consists of assessing the quality of the dataset as a proxy for observed values. This is achieved by comparing the FWI from reanalysis with the same index calculated using local data from a set of monitoring stations. Several meteorological observations are available at SYNOP stations. For the validation of FWI we used: 2 meter temperature (*T*_2_), total precipitation accumulated over the previous 24 h (*P*), 10 meter wind speed (*W*) and dew point temperature (*T*_*d*_). Relative humidity (*RH*), needed by the FWI algorithm, is calculated from *T*_2_ and *T*_*d*_ using the following equation which expresses an approximation of the inverted Clausius-Clapeyron relation^18^:

(1)RH=6.11*107.5*Td−273.15237.7+(Td−273.15)6.11*107.5*T2−273.15237.7+(T2−273.15)*100

**Figure 1:**
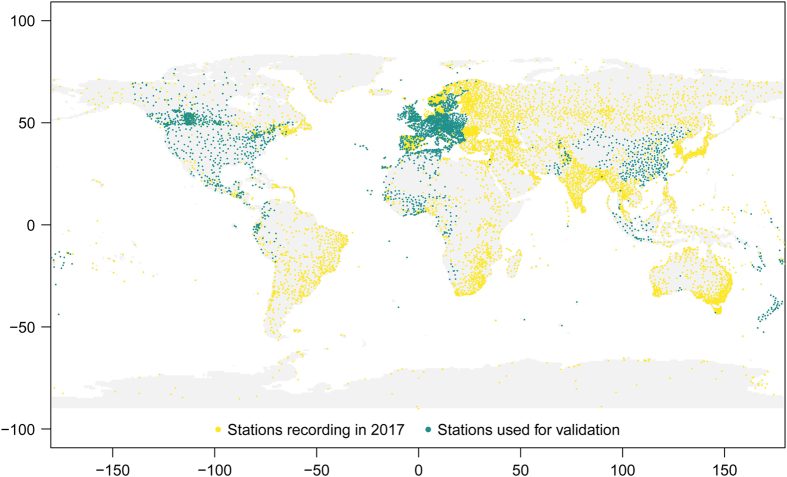
Map of SYNOP stations. Yellow dots show locations of all the stations in use in 2017. A subset of these (green dots) have enough records to be used for validation.

Although about 10,000 SYNOP stations recorded data in 2017, only 3084 of them recorded all the necessary variables between 11 and 13 local time. This time window encompasses the local noon, time at which the FWI from ERA-Interim is calculated. Comparing values within a time window, rather than at 12 local noon, was necessary to be able to extend the validation to as many stations as possible and avoid time-offsets deriving from the use of Daylight Saving Time in some countries. All the SYNOP stations and the subset used for this validation exercise are shown as yellow and green dots, respectively, in Fig. 1. Stations suitable for validation are unevenly distributed in space: 94% fall in the northern hemisphere and the remaining 6% in the southern hemisphere.

Observed and modeled FWI were compared using two metrics: bias and anomaly correlation. The bias measures how the average reanalysis magnitude compares to the average observed magnitude. The anomaly correlation compares how the modeled anomalies correspond to the observed anomalies.

**Figure 2:**
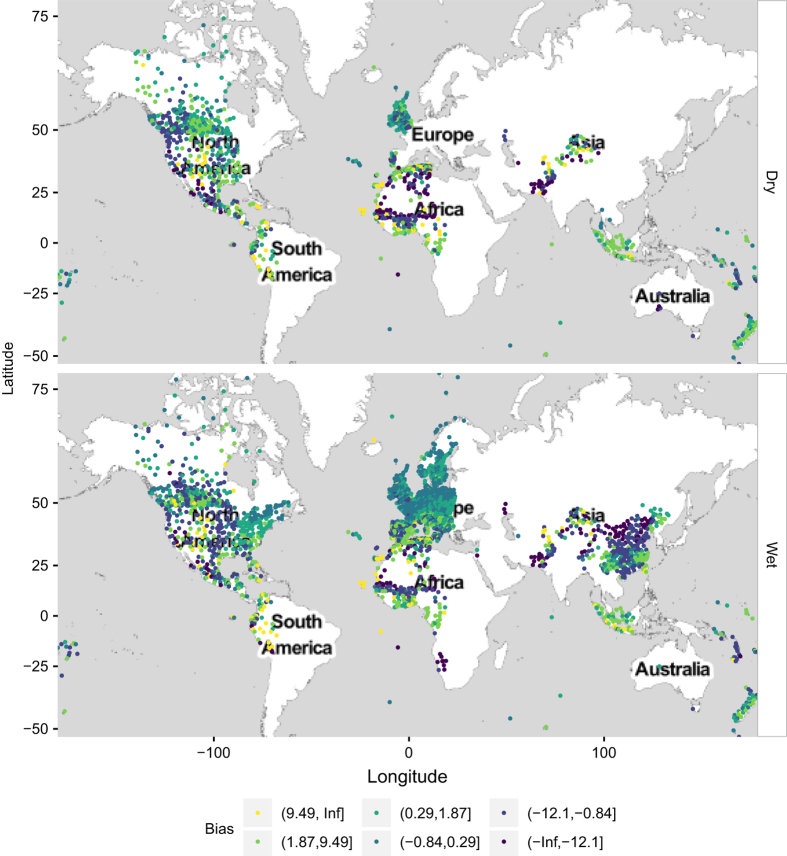
Spatial distribution of the model bias. Wet and dry season are shown in different panels.

**Figure 3:**
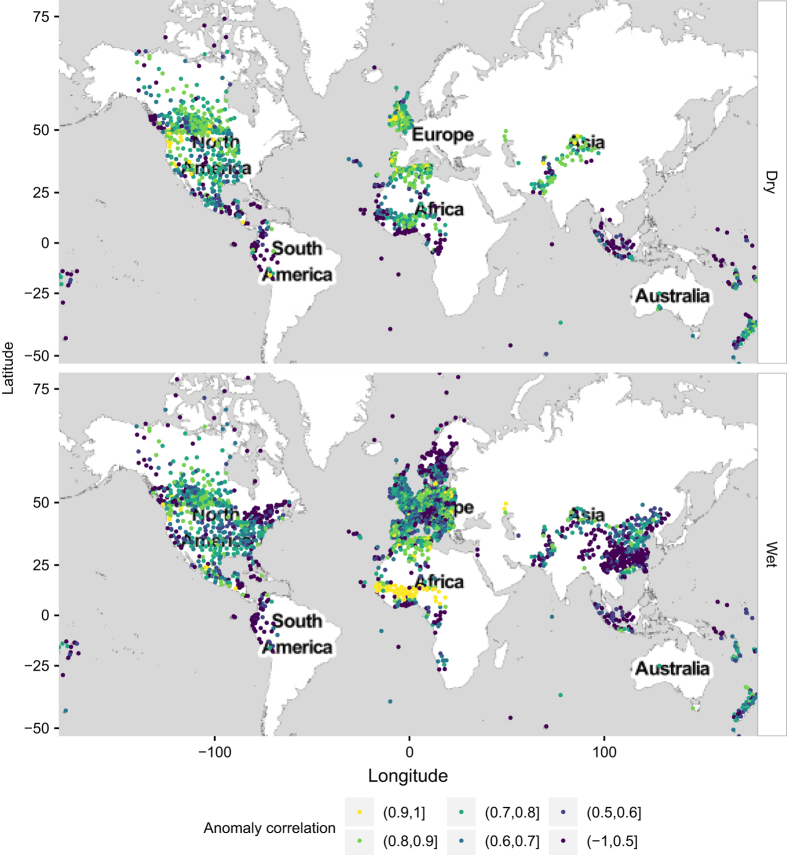
Spatial distribution of the anomaly correlation of the model. Wet and dry season are shown in different panels.

The average bias and anomaly correlation are calculated at each station, during the wet and dry seasons separately. By convention, the dry season in the northern hemisphere is assumed to start on 1st April and end on 30 September, while in the southern hemisphere it starts on 1st October and ends on 31st March. The remaining part of the year is assumed to be the wet season. Stations with less than 30 days of recordings are discarded. The spatial distribution of the bias and anomaly correlation at the SYNOP stations varies with season. This is mapped in Figs 2 and 3 and summarized in Table 2.

The metrics are mapped into 6 categories each: the bias goes from −*∞* to +*∞* with breaks corresponding to the 5th, 25th, 50th, 75th and 95th local percentiles; the anomaly correlation goes from −1 to +1 with breaks at 0.50, 0.60 (threshold commonly used to identify skillful models), 0.70, 0.80 and 0.90. Data is also distributed unevenly in time, with 70% of the records falling in the wet season and the remaining 30% in the dry season (the most relevant in the context of wildfires).

ERA-Interim performs particularly well in Europe, as a result, the FWI validation shows low biases and high anomaly correlations. The comparison between the calculation performed using ERA-Interim and the measurements recorded by the SYNOP stations is more problematic in the Arctic, Indian and Pacific oceans where the anomaly correlation is below 0.6. Problems in the representations of winds in ERA-Interim at northern latitudes are well known. For instance, surface wind speed over the Scoresby Sund region of east Greenland are lower than expected; in addition a climatology of katabatic flow along the southeast coast of Greenland demonstrates that ERA-Interim wind speed is not consistent with observations of topographically forced drainage flow in this region^19^. Tip jets and polar cyclones are additional examples of Arctic mesoscale phenomena not well represented by the global reanalyses. In the Indian and Pacific ocean, the coarse resolution of the model represents the biggest challenge for the correct representation of the land-sea climate. In fact, SYNOP observations located over islands are often not represented as land points in the ERA-Interim database because of the coarse resolution.

In North America, with the exception of the west coast, bias is small in the dry season but tends to become larger in the wet season because of the challenges of representing the timing of the frontal systems at the mid-latitudes. Another interesting aspect is the poor performances of ERA-Interim for all stations in proximity to the western coasts of continents. Inaccuracies in the precipitation representation have been improved by recent developments of the precipitation model scheme. For instance, it is worth mentioning that a new diagnostic closure introduced in the convection scheme^22^ and a new parameterization of precipitation formation^21^ are implemented in the new ECMWF reanalysis ERA-5, which is due for release in the next future.

Large positive biases also occur along the edges of the Saharan region, east Asia and central America. Stations falling in islands and coastal areas as well as in South America, West Africa and central Asia show the largest negative biases. In these zones, the metrics distributions do not change significantly with the season and its low performance is mostly due to the scarce representativeness of the grid box climate. The use of the model in these areas should, therefore, be carried out with caution. The departures from the mean are positively correlated with an average anomaly correlation above 0.60 (threshold of skillful forecast) in North America, Europe and west Africa. Lower performances are observed in South America, east and tropical Asia as well as in Scandinavia.

These results should be interpreted by taking into account the representativeness of the ERA-Interim grid-box when meteorological fields are compared to point-wise measurements. While temperature and relative humidity fields are more spatially homogeneous, precipitation and wind speed, especially during convective events, are likely to have larger spatial variations which are difficult to capture on a 80 km grid. This would justify a general smoothing of the FWI values when compared to local observations. Despite this obvious limitation, ERA-Interim dataset provides a continuous record of FWI value with a global coverage that would be simply not achievable with the current observing capabilities (see Fig. 1). As for any reanalysis dataset, the strength lies in the homogeneity and consistency of the record.

## Usage Notes

**Figure 4:**
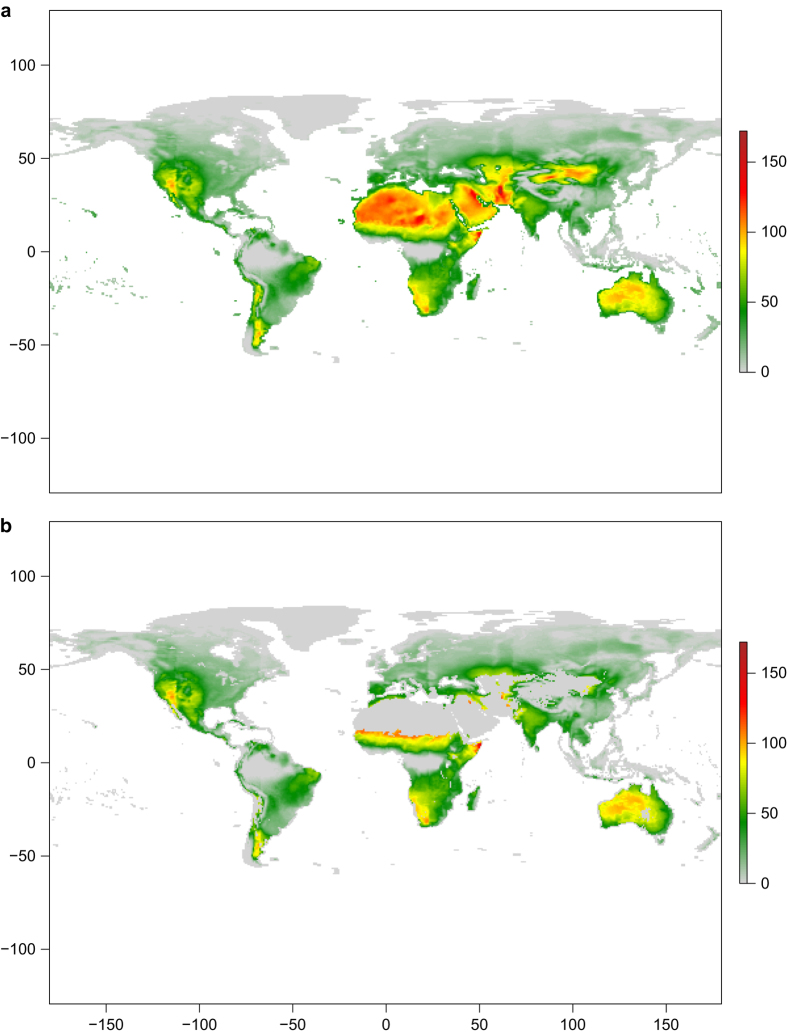
90^*th*^ percentile of FWI. Values are calculated cell by cell for the period 1980–2017. (**a**) Unmasked layer. (**b**) Masked layer.

The spatial distribution of the FWI indices varies greatly across the globe. As weather is the determining factor, even if values can be calculated everywhere, they are useful only where there is availability of fuel. Generally, before any processing, areas where vegetation is absent (bare areas, water bodies, permanent snow and ice) should be masked out. Thanks to the availability of time series global land cover maps, this is nowadays possible with a fair degree of reliability. In Fig. 4a we show the 90^*th*^ percentile of the Fire Weather Index where values are calculated cell by cell for the period 1980–2017 (data for 2018 is incomplete, available up to end of June, therefore it was discarded). In Fig. 4b we show the same layer with non vegetated areas masked out using the European Space Agency GlobCover 2009 land cover map (http://due.esrin.esa.int/page_globcover.php).

Once fire danger reanalysis data has been obtained and masked, these data can be used for a number of different purposes, some of which have been highlighted in previous sections. The typical use case is the need to bin the values into danger classes so that danger levels can be compared across places, even if characterised by climatologically different fire weather. The scientific workflow to achieve this is conceptually simple but the procedure employs numerous techniques and can be difficult to implement. For this reason, the authors of this work also implemented the R package *caliver*^9,14^ which contains functions to efficiently manipulate fire danger indices and calculate local fire danger levels. The package contains vignettes with step-by-step instructions to generate danger levels at large scale, country and regional level. The vignette named “An introduction to the caliver package”, in particular, contains also a verification of these danger thresholds using historically observed burned areas (from satellites).

A second use case is the reclassification of forecasts into categorical danger levels (using thresholds obtained from the reanalysis). This is important in the context of wildfire (and multi-hazard) Early Warning Systems (EWS) when forestry agencies monitor danger conditions. The persistence of elevated danger levels could be an influencing factor for decision makers that have the responsibility to put in place preventive measures and allocate resources on the ground to minimize risks for population and infrastructures. An example of this re-classification is described in the caliver vignette named “Verification of fire danger classes”. The same vignette also contains an example of how to validate past re-classified forecasts against observed Fire Radiative Power from satellite.

## Additional information

**How to cite this article**: Vitolo, C. *et al*. A 1980–2018 global fire danger re-analysis dataset for the Canadian Fire Weather Indices. *Sci. Data*. 6:190032 https://doi.org/10.1038/sdata.2019.32 (2019).

**Publisher’s note**: Springer Nature remains neutral with regard to jurisdictional claims in published maps and institutional affiliations.

## Supplementary Material



## Figures and Tables

**Table 1:** Comparison between FWI system indices as calculated by ECMWF and the R package *cffdrs*.

Code Source	FFMC	DMC	DC	ISI	BUI	FWI	DSR
R package *cffdrs*	87.69	7.29	17.76	10.85	7.28	9.46	1.45
ECMWF	87.70	8.54	19.01	10.80	8.49	10.10	1.63

**Table 2:** Number of SYNOP stations per region and season. For each group of stations, the median of bias and anomaly correlation are calculated.

	Region	Season	Stations	Bias	Anomaly correlation
1	Africa	Dry	251	0.36	0.76
2	Africa	Wet	267	1.47	0.82
3	America	Dry	670	0.73	0.74
4	America	Wet	874	0.20	0.67
5	Arctic	Wet	4	0.20	0.43
6	Asia	Dry	148	2.47	0.61
7	Asia	Wet	419	−0.54	0.47
8	Atlantic	Dry	26	2.18	0.43
9	Atlantic	Wet	27	3.08	0.42
10	Australia	Dry	5	−4.21	0.79
11	Australia	Wet	2	−0.77	0.76
12	Europe	Dry	168	0.27	0.83
13	Europe	Wet	1443	0.23	0.66
14	Indian	Dry	4	2.79	0.40
15	Indian	Wet	4	1.29	0.55
16	Pacific	Dry	83	1.09	0.55
17	Pacific	Wet	81	0.20	0.60
